# Supplementary material for: LDOC1 as Negative Prognostic Marker for Vulvar Cancer Patients
Source: Int J Mol Sci. 2020 Dec 5;21(23):9287. doi: 10.3390/ijms21239287 (PMC7730493; doi:10.3390/ijms21239287)
Supplement: Supplementary file 1 [file ijms-21-09287-s001.pdf]

International Journal of
Molecular Sciences

MDPI

Supplemetary file to

LDOC1 as negative prognostic marker for vulvar cancer patients

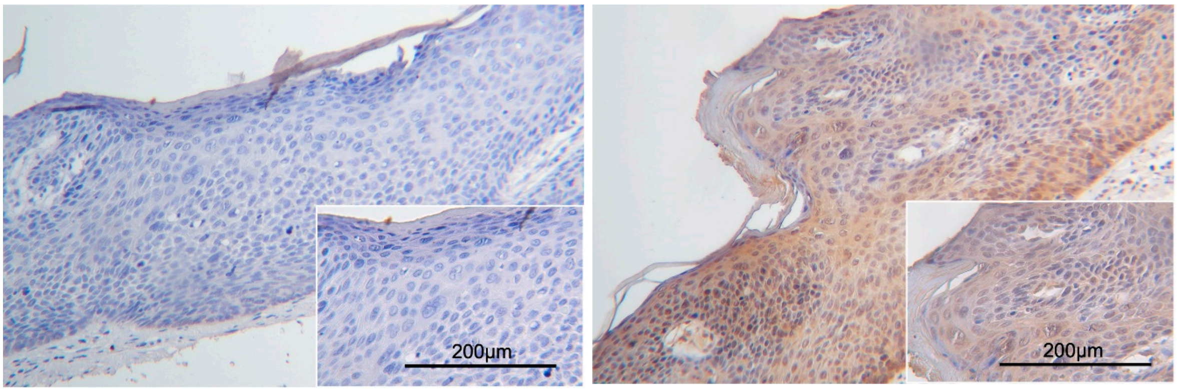

(a) **(b)**

Figure S1: Immunohistochemical staining of LDOC1 in vulvar tissue. Representative photomicrographs are presented (10× and 25× lens): **a**) no expression of LDOC1 in normal vulvar tissue; **b**) moderate cytoplasmatic expression of LDOC1 in vulvar intraepithelial neoplasia (VIN).

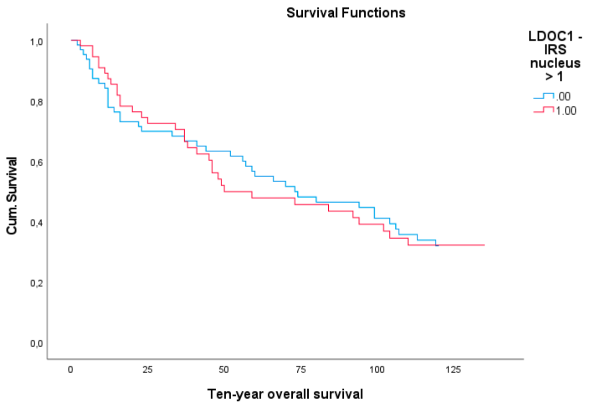

Figure S2: Kaplan-Meier survival univariate analysis for the status nuclear IRS > 1. Positive expression of LDOC1 in the cell nucleus had no influence on ten-year overall survival ($p = 0.939$).
